# Sex-specific patterns and lifetime risk of multimorbidity in the general population: a 23-year prospective cohort study

**DOI:** 10.1186/s12916-022-02487-x

**Published:** 2022-09-08

**Authors:** Premysl Velek, Annemarie I. Luik, Guy G. O. Brusselle, Bruno Ch. Stricker, Patrick J. E. Bindels, Maryam Kavousi, Brenda C. T. Kieboom, Trudy Voortman, Rikje Ruiter, M. Arfan Ikram, M. Kamran Ikram, Evelien I. T. de Schepper, Silvan Licher

**Affiliations:** 1 Department of Epidemiology, Erasmus MC - University Medical Center Rotterdam, Rotterdam, The Netherlands; 2 Department of General Practice, Erasmus MC - University Medical Center Rotterdam, Rotterdam, The Netherlands; 3 Department of Child and Adolescent Psychiatry/Psychology, Erasmus MC - University Medical Center, Rotterdam, The Netherlands; 4 Department of Respiratory Medicine, Ghent University Hospital, Ghent, Belgium; 5 Department of Respiratory Medicine, Erasmus MC - University Medical Center Rotterdam, Rotterdam, The Netherlands; 6 Division of Human Nutrition and Health, Wageningen University & Research, Wageningen, The Netherlands; 7 Department of Internal Medicine, Maasstad Hospital, Rotterdam, The Netherlands; 8 Department of Neurology, Erasmus MC - University Medical Center, Rotterdam, The Netherlands

**Keywords:** Sex differences, Multimorbidity, Risk of multimorbidity, Cohort study, Population study, Chronic diseases

## Abstract

**Background:**

Multimorbidity poses a major challenge for care coordination. However, data on what non-communicable diseases lead to multimorbidity, and whether the lifetime risk differs between men and women are lacking. We determined sex-specific differences in multimorbidity patterns and estimated sex-specific lifetime risk of multimorbidity in the general population.

**Methods:**

We followed 6,094 participants from the Rotterdam Study aged 45 years and older for the occurrence of ten diseases (cancer, coronary heart disease, stroke, chronic obstructive pulmonary disease, depression, diabetes, dementia, asthma, heart failure, parkinsonism). We visualised participants’ trajectories from a single disease to multimorbidity and the most frequent combinations of diseases. We calculated sex-specific lifetime risk of multimorbidity, considering multimorbidity involving only somatic diseases (1) affecting the same organ system, (2) affecting different organ systems, and (3) multimorbidity involving depression.

**Results:**

Over the follow-up period (1993–2016, median years of follow-up 9.2), we observed 6334 disease events. Of the study population, 10.3% had three or more diseases, and 27.9% had two or more diseases. The most frequent pair of co-occurring diseases among men was COPD and cancer (12.5% of participants with multimorbidity), the most frequent pair of diseases among women was depression and dementia (14.9%). The lifetime risk of multimorbidity was similar among men (66.0%, 95% CI: 63.2–68.8%) and women (65.1%, 95% CI: 62.5–67.7%), yet the risk of multimorbidity with depression was higher for women (30.9%, 95% CI: 28.4–33.5%, vs. 17.5%, 95% CI: 15.2–20.1%). The risk of multimorbidity with two diseases affecting the same organ is relatively low for both sexes (4.2% (95% CI: 3.2–5.5%) for men and 4.5% (95% CI: 3.5–5.7%) for women).

**Conclusions:**

Two thirds of people over 45 will develop multimorbidity in their remaining lifetime, with women at nearly double the risk of multimorbidity involving depression than men. These findings call for programmes of integrated care to consider sex-specific differences to ensure men and women are served equally.

**Supplementary Information:**

The online version contains supplementary material available at 10.1186/s12916-022-02487-x.

## Background

Multimorbidity, the co-occurrence of two or more long-term diseases, is one of the key challenges for healthcare. With improvements in survival from chronic diseases and continuous advances in their treatment, the population-level burden of disease is moving from premature mortality to long-term disability. The prevalence of multimorbidity increases rapidly with age: in high-income countries, more than 50% of people over 65 years have multimorbidity [1]. By 2035, this number is projected to increase to 68% and the gains in life expectancy will be offset by an increased number of years lived with multimorbidity [2].

Multimorbidity leads to the increased complexity of care [3]. Recommendations based on clinical practice guidelines for individual diseases often provide little guidance for people with several chronic diseases. Considering each co-existing disease separately may lead to duplication of care and increased burden of treatment [4] which is further exacerbated by increased frailty, commonly associated with multimorbidity. However, the extent of this complexity depends on the combinations of diseases that are being treated. If two co-occurring diseases affect the same organ system, for instance, they may be managed as part of a single treatment. Conversely, if they affect different organ systems, they may require separate time-intensive treatments with potential adverse drug-drug and drug-disease interactions [3, 4].

Despite growing research on multimorbidity, little is known about what diseases most frequently lead to multimorbidity and whether they differ between men and women. Previous research suggests that there is a stable set of diseases that occur in the majority of patients with multimorbidity; however, such common diseases differ between sexes and age groups [5–7]. Given the high and continuously increasing prevalence of multimorbidity, it is important to identify common patterns of multimorbidity that could guide the development and implementation of multimorbidity guidelines. A sex-specific lifetime risk for different combinations of diseases leading to multimorbidity, defined according to the type of disease (somatic vs psychiatric) and the number of organ systems affected could help better organise treatment for patients with complex needs. Lifetime risk estimates are easier to interpret than, e.g. incidence rates, and can account for risk of competing events. As such, they can be used to communicate risks to policymakers, clinicians and the public [8].

Another important aspect of multimorbidity is the interaction between depression and somatic diseases, both in terms of the combined effect on mortality, and increased complexity of care. Available evidence shows that the presence of depression in patients with multimorbidity leads to a greater decrement in health than any two or more somatic diseases [9], yet fewer than half of studies included depression in their multimorbidity measure [10].

In this study, we leveraged 23-year follow-up data from a prospective cohort study. We followed close to 15,000 individuals for ten non-communicable diseases that cause one of the highest burden of disease in older populations (nine somatic diseases and depression as a psychiatric disease). We plotted disease trajectories for each individual and examined what combinations of chronic diseases co-occur among men and women. We calculated the lifetime risk of multimorbidity for different somatic-somatic and somatic-psychiatric disease combinations, and the overall risk of multimorbidity for middle-aged individuals who are free from these diseases at baseline.

## Methods

### Study design and population

This study is embedded within the Rotterdam Study, a prospective, population-based cohort study to assess the occurrence and determinants of age-related diseases in the general population [11]. In 1990, all inhabitants aged 55 years and older from a well-defined suburb in the city of Rotterdam, the Netherlands, were invited to participate. This initial cohort comprised 7983 participants (response rate 78%). In 2000, 3011 participants aged 55 years and older were added to the cohort (response rate 67%). In 2006, 3932 participants were included, aged 45 years and older (response rate 65%). In 2016, additional 3368 participants aged 40 years and older were included (response rate 46%). In total, the Rotterdam Study comprises 18,294 participants aged 40 years or more.

We followed study participants for the occurrence of any of the following diseases: cancer (excluding non-melanoma skin cancer), coronary heart disease, stroke, chronic obstructive pulmonary disease (COPD), depression (including clinically relevant depressive symptoms), type 2 diabetes, dementia, asthma, heart failure, and parkinsonism (including Parkinson’s disease). The selected diseases are common age-related diseases that have a high burden of disease in older populations and are all in the core list of 20 diseases recommended for multimorbidity studies [10, 12]. The participants were selected from the first three cohorts of the Rotterdam Study (*n* = 14,926, 55 and older for the first two cohorts, 45 and older for the third cohort). To ensure that the ascertainment of disease is consistent across all participants, we excluded participants with a history of one (*n* = 2742) or more (*n* = 758) of the selected diseases at baseline. We further excluded 5019 participants who were incompletely screened at baseline for at least one disease of interest, and 313 participants who withdrew informed consent to data collection for a particular disease. The final population included 6094 participants. Baseline population characteristics were collected during the interviews and examinations conducted upon entering the study. Information about ancestry was obtained from whole genome sequencing, conducted as part of the Rotterdam Study [11].

### Ascertainment of diseases

The disease ascertainment at baseline and follow-up comprised of structured interviews and physical examinations by research physicians and affiliated staff at the research centre of the Rotterdam Study. Further information was collected from medical records maintained by participants’ general practitioner, letters from medical specialists, discharge reports and pharmacies, and through digital linkage to the Dutch national cancer registry and the public pathology database. During follow-up, active surveillance for the occurrence of any of the ten diseases took place with research assistants regularly verifying participants’ medical records and other sources for diagnoses of interest. Participants were invited for repeated examinations and interviews every 4 to 6 years. Diagnoses of depression, dementia and parkinsonism involved two-phase screening in which participants suspected of having the disease after the initial screening were invited for additional examination. Cancer diagnoses were confirmed based on pathology records. Diagnoses of depression included those that fulfilled DSM-IV-TR criteria (text revision of the fourth edition of the Diagnostic and Statistical Manual of Mental Disorders) as well as depressive episodes that were deemed clinically relevant, but did not meet DSM-IV-TR criteria. Final diagnoses were adjudicated by a consensus panel composed of research physicians and medical specialists affiliated with the Rotterdam Study. Disease-specific definitions, procedures, and data collection methods have been described previously, a summary is available in the Additional file 1: Ascertainment of diseases [13–24]. We defined multimorbidity as the co-occurrence of at least two diseases out of the ten diseases included in the study.

### Statistical analyses

We conducted the analyses in three steps. First, we focused on disease trajectories from a single disease to multimorbidity and visualised those trajectories for each participant in a Sankey diagram. Each participant is represented by a stripe connecting adjacent columns that represent the first three diseases in chronological order. The height of the columns is thus proportional to the number of people with each disease and in each state.

Second, we considered different combinations of co-occurring diseases recorded during follow-up, regardless of chronological order. We plotted these combinations in an intersection diagram to visualise combinations of co-occurring diseases and the number of participants with individual diseases and disease combinations [25].

Third, we calculated the lifetime risk of multimorbidity for participants aged 45 years and older, adjusted for age (as time scale). As screening for each of the ten selected diseases did not start on the same date, the follow-up start for individual participants was set to the participant’s age corresponding to the date on which follow-up data for all selected diseases were available. The event of interest was the diagnosis of the second disease in chronological order. The follow-up ended either at the age of diagnosis of the second disease, age of death, the age of loss to follow-up, or the age of the earliest end of follow-up among the selected diseases, whichever came first. Participants were considered lost to follow-up if their medical records could not be accessed. In this analysis, we defined three classes of multimorbidity, based on individual combinations of the first two diseases that led to multimorbidity. All combinations of diseases that involved depression were classified as somatic-psychiatric multimorbidity as the most diverse pair of diseases [26]. We then classified all other combinations as somatic multimorbidity. We further distinguished between multimorbidity only involving diseases affecting one organ system (somatic concordant) and multimorbidity involving somatic diseases affecting different organ systems (somatic discordant) [27]. Somatic concordant multimorbidity involved the combinations of COPD and asthma, coronary heart disease and heart failure, and parkinsonism and dementia. All other pairs of individual diseases were classified as somatic discordant. We used multi-state models to calculate the specific absolute risk for each of the three multimorbidity classes separately for men and women, accounting for the competing risk of death [28]. Finally, we calculated the overall risk of multimorbidity as the composite risks of the three multimorbidity classes.

We conducted five sensitivity analyses to assess whether our definition of multimorbidity classes affects the results. First, we grouped together dementia and depression as mental health diseases, following the International Classification of Diseases. We defined two corresponding classes of multimorbidity: multimorbidity involving mental health disease (any combination with depression or dementia), and multimorbidity involving only physical diseases (any other combinations) [10]. Second, we grouped together diseases that would be typically managed by one specialist to assess the potential risk of fragmentation of care: we grouped stroke together with parkinsonism and dementia (diseases managed by a neurologist or geriatrician); multimorbidity involving any combination of these three diseases was classified as somatic concordant. Third, we considered the combination of the first three diseases (rather than two) in our definition of multimorbidity classes to assess how many participants change their multimorbidity class when the third disease is added. Fourth, we used a stricter definition of depression and considered only DSM-IV-TR defined cases of depressive disorder (single or recurrent), dysthymic disorder, mood disorder due to medical condition and mood disorder not otherwise specified. Participants with clinically relevant depressive symptoms were considered free of depression. Finally, in the fifth analysis, we included in the study population also participants with one prevalent disease and recalculated the lifetime risk of multimorbidity.

The study was carried out following the STROBE (Strengthening the Reporting of Observational Studies in Epidemiology) guidelines. All analyses were performed in R, version 4.0.4.

## Results

We analysed data from 6094 community-dwelling individuals; there were 2364 individuals included in the first Rotterdam Study cohort, 1436 individuals in the second cohort and 2294 individuals in the third cohort. The median age at baseline was 66.0 in the first cohort, 61.3 in the second cohort and 55.9 in the third cohort.

The overall median age was 60.8 years (interquartile range (IQR): 57.1–66.7), 57.6% were women and 97.9% of the population was of European ancestry. The study period for this work started on 10 September 1993 (earliest follow-up start for an individual participant) and ended on 1 January 2016 (latest follow-up end for an individual participant). The characteristics of the study population, stratified by sex are shown in Table 1. During 63,130 years of follow-up (median: 9.2 years, IQR: 7.1–13.4), we observed 6334 disease events. A total of 1897 (31.1%) participants died during follow-up; of whom 169 (2.8%) died free of any of the selected diseases; 692 (11.1%) died with one disease, and 1036 (17.0%) died with two or more diseases. Of the observed disease events, 1084 participants screened positive for depression (330 with depressive syndromes or depressive disorders, 754 with clinically relevant depressive symptoms; of those with depressive symptoms 138 screened positive for depressive syndromes or disorders two or more years after the initial diagnosis), 1004 with cancer (of whom 118 were diagnosed with secondary cancer), 967 with COPD, 686 with diabetes, 668 with dementia, 654 with coronary heart disease, 539 with heart failure, 450 with stroke, 158 with asthma, and 124 with parkinsonism.

**Table 1:** Characteristics of the study population and the median age of the diagnosis of the first two diseases

	All participants (***n*** = 6094)	Men (***n*** = 2585, 42.4%)	Women (***n*** = 3509, 57.6%)	***p***-value for sex differences^1^
**Age (years), median (IQR)**	**60.8 (57.1–66.7)**	**60.4 (56.7–65.9)**	**61.1 (57.3–67.5)**	< 0.001
**Years of follow-up, median (IQR)**	9.2 (7.1–13.4)	9.3 (7.2–12.7)	9.1 (7.0–13.8)	0.58
**Marital status**				< 0.001
Living with partner	**4619 (80.3%)**	**2244 (90.4%)**	**2375 (72.8%)**	
Living without partner	**1127 (19.6%)**	**239 (9.6%)**	**888 (27%)**	
**Education**				< 0.001
Primary	**636 (10.4%)**	**210 (8%)**	**426 (14%)**	
Lower secondary	**2462 (40.5%)**	**703 (30%)**	**1759 (55%)**	
Further secondary	**1189 (19.5%)**	**724 (38%)**	**465 (24%)**	
Higher	**1782 (29.3%)**	**938 (23%)**	**844 (7%)**	
**Smoking status**				< 0.001
Never	**1945 (32.2%)**	**461 (17.8%)**	**1484 (42.9%)**	
Former	**2690 (44.5%)**	**1396 (53.2%)**	**1294 (37.4%)**	
Current	**1410 (23.0%)**	**719 (27.9%)**	**682 (19.7%)**	
**Blood pressure** (mm Hg), median (IQR)				
Systolic	135 (122–148)	136 (124–149)	134 (120–148)	< 0.001
Diastolic	79 (72–86)	80 (73–87)	78 (71–86)	< 0.001
**Ancestry**				0.8
European	5378 (97.9%)	2280 (98.0%)	3098 (97.9%)	
East-Asian	63 (1.1%)	28 (1.2%)	35 (1.1%)	
African	37 (0.7%)	13 (0.6%)	24 (0.8%)	
Admixed	13 (0.2%)	5 (0.2%)	8 (0.3%)	
**Age at diagnosis (years), median (IQR)**^**2**^				
First disease	**71.9 (64.5–79.0)**	**71.0 (64.0–77.6)**	**72.9 (64.9–80.3)**	< 0.001
Second disease	**78.1 (71.8–84.0)**	**77.0 (71.1–82.7)**	**79.0 (72.6–84.9)**	< 0.001

Key sex differences in the population characteristics are highlighted in bold

*IQR* interquartile range

Data at baseline were near complete (<3% missing), with the exception of the ancestry characteristic which had 9.8% of missing data (missing data were excluded when calculating the proportions)

^1^*p*-values were calculated using Wilcoxon-Mann-Whitney rank sum for continuous variables (with the null hypothesis of equal medians) and chi-squared test for categorical variables

^2^The values were calculated excluding participants who were censored or died without any diagnosis (for the age at the diagnosis of the first disease) or who were censored or died with only one diagnosis (for the age at the diagnosis of the second disease)

### Multimorbidity trajectories

**Fig. 1:**
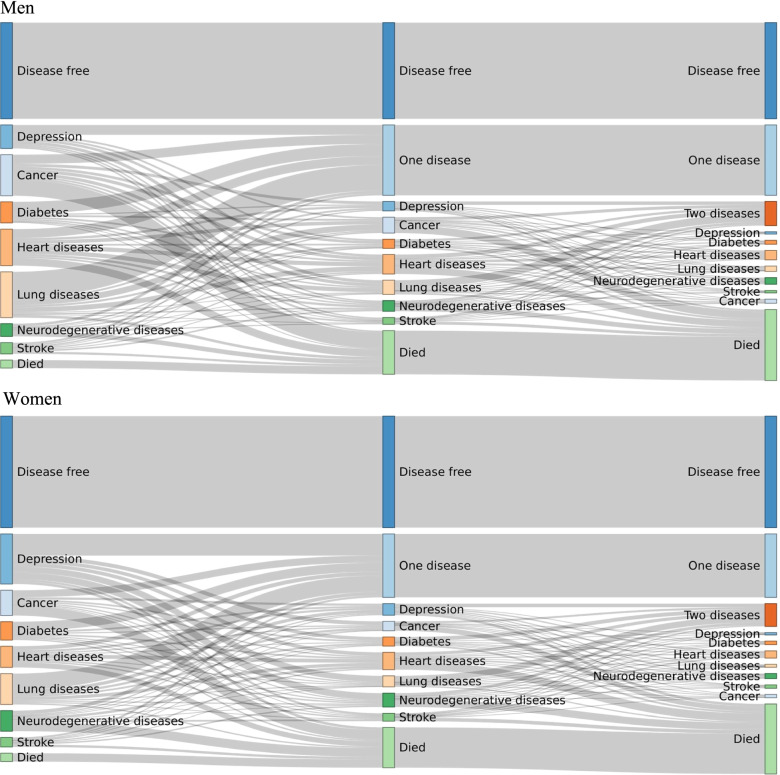
Disease trajectories from single disease to multimorbidity for men and women. The columns represent the diagnosis of first three non-communicable diseases in chronological order (from left to right). Each participant is represented by a stripe, the height of the columns and the thickness of the stripes are proportional to the number of people with a particular disease. For ease of visualisation, we grouped together diseases that affect the same organ system: neurodegenerative diseases (parkinsonism and dementia), heart diseases (coronary heart diseases and heart failure) and lung diseases (COPD and asthma); all the other diseases are represented separately. If an individual trajectory connects the same group (e.g. from heart diseases to heart diseases), then it connects different diseases within this group (i.e. coronary heart disease and heart failure, it does not imply a recurrent event). We did not consider a possible recovery from a disease, i.e. a participant cannot revert to a disease-free state or from having two diseases to having one disease. Online version of the figure shows the disease trajectories both separately for each sex and combined across sexes, and allows highlighting disease trajectories involving a particular disease selected by user https://www.ergo-onderzoek.nl/multimorbidity-velek-etal

In Fig. 1 (interactive version is available online https://www.ergo-onderzoek.nl/multimorbidity-velek-etal), we plotted disease trajectories for each participant from the onset of a single disease to multimorbidity. Of the study population, 27.9% were diagnosed with two or more diseases during follow-up, of whom 37.0% (10.3% of the total population) had three or more diseases. The maximum number of co-occurring diseases was seven, which occurred in two participants. In contrast, 35.5% of participants remained free from any of the diseases of interest during the follow-up, and 33.8% had one disease. We observed a greater proportion of women with depression as their first disease than men (odds ratio (OR) 2.82, 95% confidence intervals (CI): 2.38–-3.36). Conversely, men were more frequently diagnosed with cancer than women (OR 1.62 (95% CI: 1.37–1.92) or COPD (OR 1.75 (95% CI: 1.48–2.07) as their first disease.

### Sex-specific patterns of multimorbidity

**Fig. 2:**
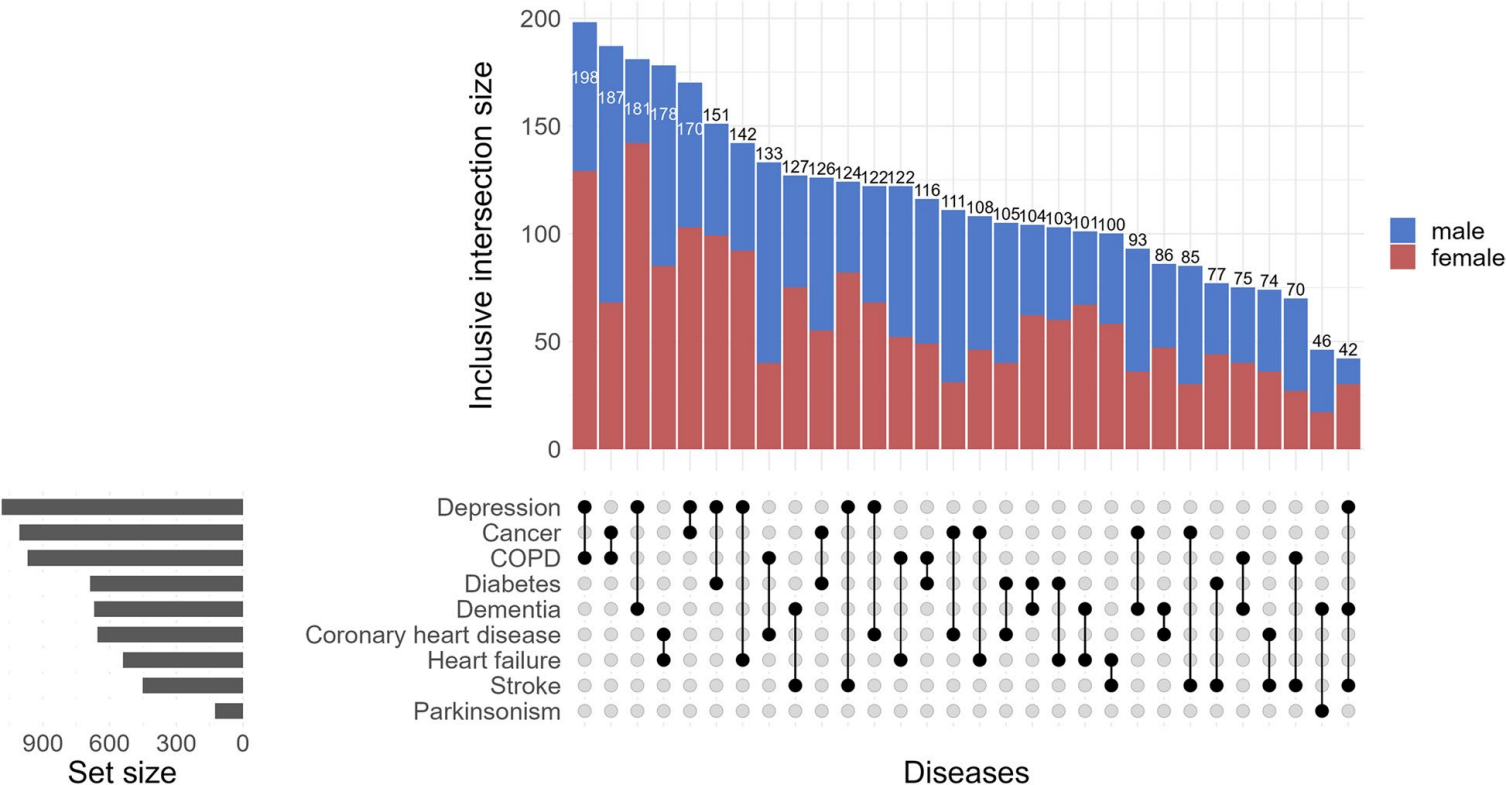
Intersection diagram with patterns of co-occurrence of diseases within single individuals. The diagram visualises co-occurrence of diseases as a matrix in which the rows represent the individual diseases and the columns represent their intersections, i.e. the different combinations of co-occurring diseases. All diseases that are part of a given combination are shown as black dots connected with a vertical black line (if a disease is not part of the combination a grey dot is shown.) The number of participants with a given combination of diseases is shown as a vertical bar on top of the matrix, the number of participants with any one disease is shown as a horizontal bar to the left of the matrix. Online version of the figure allows users to select particular combinations of diseases, involving any specific disease and includes separate plots for men and women https://www.ergo-onderzoek.nl/multimorbidity-velek-etal

The most frequent combinations of co-occurring diseases are shown in Fig. 2 (interactive version is available online https://www.ergo-onderzoek.nl/multimorbidity-velek-etal). The most common pair of co-occurring diseases is COPD and depression (11.7% participants with multimorbidity). Of the top ten most frequent combinations of diseases, five include depression (in combination with COPD, dementia, cancer, diabetes and heart failure) and three include cancer (with COPD, depression and diabetes). Among men, the most common pair of diseases is cancer and COPD (12.5% participants with multimorbidity). Of the top ten most frequent combinations, five include COPD (with cancer, coronary heart disease, hear failure, depression and diabetes), four cancer, and three coronary heart disease and diabetes. Among women, the most common pair of diseases is depression and dementia (14.9% participants with multimorbidity). Seven out of ten most frequent combinations include depression (with dementia, COPD, cancer, diabetes, heart failure, stroke and coronary heart disease).

### Lifetime risk of multimorbidity

**Fig. 3:**
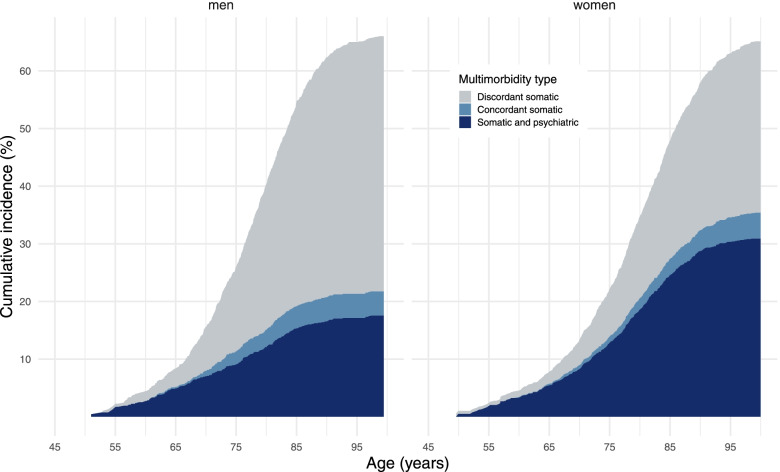
Lifetime risk of multimorbidity for men and women over 45 years free of the ten selected diseases at baseline. In this analysis, follow-up ended at the time of diagnosis of the second medical disease of interest, at the time of death, or at the time of censoring or at the end of the administrative study period. The three types of multimorbidity are based on the combinations of the first two diseases in chronological order. All combinations of diseases that involve depression were classified as somatic-psychiatric multimorbidity; all other combinations as somatic multimorbidity. Combinations of somatic diseases that affect the same organ system were classified as somatic concordant, combinations of somatic diseases affecting different organ systems were classified as somatic discordant. Somatic concordant multimorbidity involved the combinations of COPD and asthma, coronary heart disease and heart failure, and parkinsonism and dementia. All other pairs of individual diseases were classified as somatic discordant

The overall lifetime risk of multimorbidity for men in our sample is 66.0 % (95 CI: 63.2–68.8%) and 65.1% (95% CI: 62.5–67.7%) for women in our sample (*p*-value for the sex difference 0.33). The risk of multimorbidity increases steeply with age: from 8.5% (95% CI: 6.5–10.9%) at the age of 65 years for men and 7.9% (95% CI: 6.0–10.5%) for women, up to 26.1% (95% CI: 23.5–28.9%) for men and 22.2% (95% CI: 19.9–24.9%) for women at the age of 75 years. The estimated lifetime risk of multimorbidity involving depression (somatic-psychiatric multimorbidity) is almost twice as high for women as for men (30.9% (95% CI: 28.4–33.5%) vs. 17.5% (95% CI: 15.2–20.1%), *p*-value < 0.001). Conversely, men have higher lifetime risk of multimorbidity with two somatic discordant diseases (44.2% (95% CI: 41.3–47.2%) vs. 29.7% (95% CI: 27.3–32.1%), *p*-value < 0.001). Compared with somatic discordant and somatic-psychiatric multimorbidity, the lifetime risk of somatic concordant multimorbidity (i.e. a combination of disease affecting the same organ system) remain relatively low (4.2% (95% CI: 3.2–5.5%) for men and 4.5% (95% CI: 3.5–5.7%) for women, *p*-value for sex difference 0.35) (Fig. 3).

Sensitivity analyses that involved alternative grouping of diseases and alternative definition of our multimorbidity classes resulted in increased risk of somatic concordant multimorbidity, but did not change substantially the sex-specific differences. The lifetime risk of multimorbidity dropped when we excluded cases of clinically relevant depressive symptoms (from 66.0 to 61.2% for men and from 65.1 to 55.7% for women). Yet, the relative difference between men and women in the risk of somatic-psychiatric multimorbidity was similar (12.0% (95% CI: 10.3–13.9) for women vs 6.4% (95% CI: 5.0–8.1) for men). Including participants with one prevalent disease at baseline, increased the overall risk of multimorbidity but the sex-specific differences pertaining to somatic-psychiatric multimorbidity did not change (17.9% (95% CI: 16.4–19.7%) for men and 33.9% (95% CI: 32.1–35.9%) for women). See Additional file 2: Sensitivity analysis for complete results.

## Discussion

We demonstrated that nearly two thirds of the population over 45 years will develop multimorbidity in their remaining lifetime. Importantly, the patterns of co-occurring diseases differ substantially between men and women. Whereas the most frequent combinations of diseases for men involve COPD and cancer, the most frequent combinations of diseases for women involve depression. Consequently, the lifetime risk of multimorbidity with depression is nearly twice as high for women as for men (30.9 vs.17.5%), and the lifetime risk of multimorbidity with two discordant somatic diseases is considerably higher for men than women (44.2 vs. 29.7%). The lifetime risk of multimorbidity with two concordant somatic diseases does not exceed 5% for both sexes.

Our results show that multimorbidity affects men and women to a similar extent and the majority of multimorbid patients have a combination of diseases that affect different organ systems. Furthermore, women are at much higher risk of multimorbidity with depression and the difference increases with age. In health care systems with no requirements for referrals from primary care, multimorbidity with discordant diseases leads to higher use of different specialist care, even for patients who are not considered to require one [29]. This fragmentation may have serious consequences for continuity of care and directly leads to increased burden of treatment. In countries with comprehensive primary care (e.g. the UK or the Netherlands), the risk of fragmentation is lower as general practitioners manage most stable patients with multimorbidity. However, even in the primary care setting, interactions between different co-occurring diseases and synergies in their management are not routinely considered [30] and several models for integrated care have been proposed [31, 32]. Managing patients with multimorbidity involving depression, adds additional challenges: depression in combination with another somatic disease worsens patients’ health more than any one or more diseases [9]. Moreover, management of multimorbid patients with depression is often geared towards somatic diseases and those patients are less likely to receive prescriptions or referral as compared to those with only depression [33]. It is likely that the underlying cause behind sex differences in the risk of multimorbidity with and without depression is multifactorial. A possible mechanism may lie in the potential sex differences in the sensitivity to stress which may lead to a higher risk of depression but also to a higher prevalence of multimorbidity with depression among women [34].

The series of our sensitivity analyses showed that our results are robust to different alternative definitions of multimorbidity classes, suggesting that there are consistent differences between men and women in multimorbidity patterns. When using more strict diagnostic criteria for depression, the absolute risk of somatic-psychiatric multimorbidity decreased, but the relative difference between men and women persisted. Nevertheless, there is evidence that depressive symptoms have considerable effect on well-being and that the mortality rate in depressive symptoms is comparable to the rate in major depression [35]. Including depressive symptoms is therefore likely to provide a more complete picture of the burden of multimorbidity.

The relatively low lifetime risk of concordant multimorbidity in both men and women suggests that in the majority of cases, multimorbidity leads to greater complexity of care with risk of polypharmacy and adverse drug interactions. Potentially serious drug-drug interactions were found to be common for a range of diseases co-occurring with depression, type 2 diabetes and heart failure, even for combinations of diseases that share risk factors [36]. The risk of adverse drug-drug interaction therefore poses an additional challenge in managing people with multimorbidity, regardless of sex.

### Previous research

In line with previous research, we found that both the risk of multimorbidity and the differences between men and women concerning multimorbidity with mental health diseases increased rapidly with age [1, 26]. Our estimates of the overall risk of multimorbidity are lower than existing estimates of prevalence for corresponding age groups; we also did not observe a higher risk of overall multimorbidity among women, as reported by some studies [1]. This may be due to the different number and type of diseases considered, as we did not include diseases with a low burden of treatment (such as hypertension) and locomotor diseases with high prevalence among elderly (such as osteoarthritis). Our finding that women have a higher absolute risk of multimorbidity with depression is in line with a US study on incident multimorbidity that found that women have a higher risk of depression than men across all ages and a higher risk of multimorbidity with depression [7]. Another US research observed 1.7 higher prevalence of multimorbidity involving a mental health disease among women [26]. A systematic literature review of disease clusters in older adults identified depression among the top twelve diseases that co-occur with other diseases most often [6]. A cross-sectional study from Canada found that non-psychotic mood and anxiety disorders co-occur more frequently with other diseases among middle-aged women than among men [5]. A cohort study from the USA showed that middle-aged and older women (but not men) with depression and/or anxiety have a higher risk of accumulating other chronic conditions as compared to women without depression or anxiety [34]. A Danish study on the effect of prior mental health disease on subsequent physical diseases found a higher risk for women than men for pulmonary and neurological diseases but not for circulatory diseases [37]. Our finding regarding the relatively low risk of multimorbidity with two concordant somatic diseases confirms previous results. The most frequent and most prevalent pairs of co-occurring diseases identified by other studies predominantly involve diseases affecting different organ systems [5, 6], although different selection, definition and ascertainment of diseases across studies limit direct comparison of results.

### Limitations and strengths

The major strength of the study includes continuous, long-term follow-up in a single population, which enabled us to overcome the limitations of studies that rely solely on electronic health records, such as inconsistent recording or detection bias. Consistent methods of disease ascertainment combining medical files with interviews and physical examination minimised the risk of misclassification and enabled us to identify participants’ trajectories from a single disease to multimorbidity and estimate the absolute risk of multimorbidity. 

There are several limitations of this study. First, it is possible that non-responders to the invitation to participate in the Rotterdam study (28%), participants with incomplete data (34%) or participants with two or more prevalent diseases at baseline (5%) may have had a higher risk of multimorbidity which would lead to underestimation of our results (see Additional file 3: Table A1, A2 and A3 for population characteristics of excluded participants). Second, we did not have continuous follow-up available on chronic kidney disease, liver diseases, chronic pain, sensory impairments or osteoarthritis, which largely affect older adults; we also did not have data on other types of psychiatric diseases such as anxiety or personality disorder. Including these diseases would likely lead to a higher lifetime risk of multimorbidity. Depression is thought to be underreported in men [38]. Potentially, this could have artificially increased the sex differences in multimorbidity with depression. Within the Rotterdam Study, depression is assessed at each research visit with validated questionnaires and interviews in addition to participants’ medical records to minimise underreporting as much as possible. Third, we did not consider recurrent stroke or secondary cancer as separate diseases. Including these data would increase the risk of multimorbidity, though the increase would be relatively small: the risk of secondary cancer in our study population is 11.8%, and a recent estimate of 10-year risk of stroke recurrence is 13% [39]. Finally, as the study participants have predominantly European ancestry, findings have limited generalisability to other ethnicities.

## Conclusions

Our results show that depression is the most frequently occurring disease among women with multimorbidity, whereas the most common diseases among men with multimorbidity are COPD and cancer. Consequently, women have close to twice as high lifetime risk of multimorbidity involving depression than men (30.9 vs.17.5%), whereas men have a higher risk of multimorbidity with somatic diseases (44.2 vs. 29.7%). There is a risk that women are disproportionally affected by suboptimal care resulting from the additional challenges posed by the presence of depression in multimorbid patients. Many countries are now implementing programmes of integrated care aimed at patients with multimorbidity and there is an ongoing debate about what interventions are the most effective [40]. Our findings support the need for a sex-specific approach in prevention and care management, based on the most frequent clusters of co-occurring diseases in men and women. It is crucial that such programmes consider multimorbidity with psychiatric diseases to ensure that both men and women equally benefit from them.

## Supplementary Information

**Additional file 1.** Ascertainment of diseases. A summary of methods of ascertainment of the ten diseases selected diseases.

**Additional file 2. **Sensitivity analysis. Results of the five sensitivity analyses presented.

**Additional file 3. **Population characteristics of excluded participants.

**Additional file 4. **STROBE reporting checklist for cohort studies.

## Data Availability

The datasets generated and/or analysed during the current study are not publicly available due to legal and ethical restraints but are available upon reasonable request. Requests can be directed to secretariat.epi@erasmusmc.nl or visit the following website for more information http://www.ergo-onderzoek.nl/wp/contact. Sharing of individual participant data was not included in the informed consent of the study, and there is a potential risk of revealing participants’ identities as it is not possible to completely anonymize the data.

## Abbreviations

COPD: Chronic obstructive pulmonary disease
DSM-IV-TR: Text revision of the fourth edition of Diagnostic and Statistical Manual of Mental Disorders
CI: Confidence intervals
OR: Odds ratio
STROBE: Strengthening the Reporting of Observational Studies in Epidemiology
ZonMw: Netherlands Organization for Health Research and Development
IQR: Interquartile range
