# Supplementary material for: Sex-specific patterns and lifetime risk of multimorbidity in the general population: a 23-year prospective cohort study
Source: BMC Med. 2022 Sep 8;20:304. doi: 10.1186/s12916-022-02487-x (PMC9454172; doi:10.1186/s12916-022-02487-x)
Supplement: Supplementary file 1 — Additional file 1. Ascertainment of diseases. A summary of methods of ascertainment of the ten diseases selected diseases. [file 12916_2022_2487_MOESM1_ESM.docx]

**Additional file 1: Ascertainment of diseases**

Additional file for article Velek, P, Luik AI, Brusselle GGO, *Sex-specific patterns and lifetime risk of multimorbidity in the general population: a 23-year prospective cohort study*

### **Ascertainment of diseases**

#### **Cancer**

Both prevalent and incident cases were based on medical records from general practitioners (including hospital discharge letters) and through linkage with the national hospital discharge registry (Landelijke Medische Registratie) and with cytopathology registries in the region (part of the nationwide network PALGA).

Cancer diagnosis was coded independently by two physicians and classified according to the International Classification of Diseases, 10th revision (ICD‐10). Only pathology confirmed cancer diagnoses were used; non-melanoma skin cancers were excluded. Date of diagnosis was based on the date of biopsy (solid tumors), or laboratory assessment (hematologic tumors). In case of discrepancy between sources, consensus was sought through consultation with a physician specialised in internal medicine.(13)

#### **Coronary heart disease and heart failure**

Prevalent cases were based on clinical information on the history of cardiovascular events obtained from the general practitioners. Additional information was obtained from the hospitals if needed. Furthermore, upon entering the study, each participant was interviewed on the occurrence of cardiac events in the past and examined at the Rotterdam Study research centre.

Incident cases were based on medical records from general practitioners (including discharge letters) and repeat interviews and examinations at the Rotterdam Study research centre.

The diagnosis of coronary heart disease includes the following outcomes: myocardial infarction, unrecognized myocardial infarction, coronary heart disease mortality and overall coronary heart disease. (14) The diagnosis of heart failure was determined by using a validated score, based on the presence of at least two signs or symptoms suggestive of heart failure: shortness of breath, ankle swelling and pulmonary crepitations; or use of medication for the indication of heart failure, in combination with objective evidence of cardiovascular disease. (15)

For each outcome (prevalent and incident) two cardiovascular research physicians independently classified information on occurrence, certainty, and date of onset of all data collected on potential events. Cases on which the research physicians disagreed were discussed in order to reach consensus in a separate session. Afterwards, a panel of medical specialists reviewed potential events for each diagnosis separately. The medical specialist’s judgment was considered decisive. The research physicians and the medical specialists base their decisions on the same data.

#### **Stroke**

Information about prevalent cases was obtained from interview at baseline and verified based on medical records from general practitioners. Incident cases were based on continuous monitoring of participants’ medical files for possible cases of stroke and repeat interviews and examinations at the Rotterdam Study research centre.

Stroke was defined according to the World Health Organization (WHO) criteria as a syndrome of rapidly developing clinical signs of focal (or global) disturbance of cerebral function. Symptoms had to last 24 hours or longer or leading to death, with no apparent cause other than vascular origin. (16) Strokes were further classified as cerebral infarction or intracerebral hemorrhage based on neuroimaging reports. If neuroimaging was lacking, a stroke was classified as unspecified. Transient ischemic attacks or subarachnoid hemorrhages were not included. Research physicians reviewed potential cases of strokes, and a consensus panel led by a consultant neurologist verified and agreed on all diagnoses of stroke. (17(

#### **Chronic obstructive pulmonary disease (COPD) and asthma**

COPD was diagnosed by an obstructive spirometry (FEV_1_/FVC < 70%) performed at the Rotterdam Study research centre. In absence of an interpretable study-acquired spirometry, the medical records were reviewed of all patients who regularly used medication for obstructive lung disease (Anatomical Therapeutic Chemical Classification codes: R03). Each such potential case was subsequently validated through careful evaluation of all medical records, hospitalizations and specialist letters and only included if a clear and well-founded diagnosis of COPD was retained. (18)

Asthma cases were defined as participants with a physician's diagnosis of asthma reported in their medical file. The identification of subjects with a diagnosis of asthma consisted of two steps: case finding and validation. The case finding phase bundled all participants with either: ever asthma and/or respiratory complaints in the questionnaire, the use of respiratory medication, an obstructive pulmonary function test, asthma as reported in the patient's medical file, hospital admissions for asthma, or a death record that reported asthma. Next, the medical files of all these possible asthma cases were systematically reviewed to search for asthma as diagnosed by a physician. Cases of asthma were defined as diagnosed by either a pulmonary physician/ asthma allergist or by a GP (as evidenced in the medical files). (19)

The interpretation of the medical records and the spinometry results was performed independently by two research physicians; in case of discordance, the final protocol was made by a senior respiratory physician.

#### **Depression**

At baseline, all participants filled out either the Dutch versions of the Center for Epidemiologic Studies Depression Scale (CES-D) or the Dutch version of the Hospital Anxiety and Depression Scale. A score of 16 or higher on the CES-D was considered indicative of a depressive disorder; a score of 9 or higher on the Hospital Anxiety and Depression Scale was considered as the cutoff for depression. Cases of depression identified at baseline were considered prevalent.

During the repeat examinations, all participants were screened with the CES-D as part of the home interview. The screen-positive participants were invited for a clinical interview conducted by a psychiatrist, psychogeriatrician, or clinical psychologist. With a computerized diagnostic algorithm based on the item scores, major and minor depressive disorders and dysthymia were classified according to DSM-IV-TR criteria. Active surveillance for the occurrence of depression took place during the follow-up, using a number of predefined cues such as symptoms of depression, prescriptions of psychiatric medication, the occurrence of major life events, and psychosocial problems.

Two categories of depression were defined: depressive syndromes, including DSM-IV-TR depressive disorders and bipolar disorder, and clinically relevant depressive symptoms. The category of depressive syndromes consisted of major depressive disorder (MDD) and dysthymia together and other depressive syndromes. The group of other depressive syndromes covered the following: (1) depression recorded by a GP or physician; (2) self-reported depression for which the participant consulted a GP or a mental health professional; and (3) DSM-IV minor depression. The category of clinically relevant depressive symptoms included the following: (1) clinically relevant core symptom of major depression recorded during the psychiatric interview or in the medical record; (2) self-reported depression of a participant who did not consult a GP or a mental health professional; and (3) initiation of antidepressant drug treatment (without documentation of clinical symptoms). Grief, adjustment disorder, and burnout, characterized by emotional exhaustion and reduced satisfaction in personal accomplishment were not regarded as depression.

All information that indicated potential depression was reviewed by two physicians and a research psychologist, a final diagnosis was determined at consensus meetings. (20)

#### **Type 2 Diabetes**

Both prevalent and incidence cases of type 2 diabetes were ascertained by use of GP’s records (including laboratory glucose measurements), hospital discharge letters, and serum glucose measurements performed at baseline and repeat physical examination at the Rotterdam Study research centre. Type 2 diabetes was defined as a fasting blood glucose concentration of 7.0 mmol/L or higher, a non-fasting blood glucose concentration of 11.1 mmol/L or higher (when fasting samples were unavailable), or the use of blood glucose-lowering drugs. Information about the use of blood glucose-lowering drugs was obtained from both structured home interviews and pharmacy dispensing records.

All potential cases of type 2 diabetes were independently adjudicated by two study physicians. In case of disagreement, consensus was sought from an endocrinologist. (21)

#### **Dementia**

Participants were screened for dementia at baseline and subsequent center visits with the Mini-Mental State Examination and the Geriatric Mental Schedule organic level. Those with a Mini-Mental State Examination score < 26 or Geriatric Mental Schedule score > 0 underwent further investigation and informant interview, including the Cambridge Examination for Mental Disorders of the Elderly. All participants also underwent routine cognitive assessment.

During follow-up participants were continuously under surveillance for dementia through electronic linkage of the study database with medical records from general practitioners and the regional institute for outpatient mental health care.

A consensus panel led by a consultant neurologist established the final diagnosis according to standard criteria for dementia (DSM-IIIR), Alzheimer's disease (NINCDS–ADRDA) and vascular dementia (NINDS-AIREN). (22)

#### **Parkinsonism**

A two-phase design was used to identify both prevalent and incident cases of parkinsonism or Parkinson’s Disease (PD). At baseline and during repeat physical examination at the Rotterdam Study research centre, all subjects were asked about previous diagnosis of PD, and any drug use was coded according to the Anatomical Therapeutic Chemical (ATC) classification index. (23) In addition, every participant was neurologically examined by one of the research physicians. All subjects who either used antiparkinsonian drugs (ATC code N04), reported that they had PD, or had at least one possible cardinal sign of parkinsonism (i.e., resting tremor, cogwheel rigidity, hypo- or bradykinesia, or impaired postural reflexes) at the neurologic screening examination were invited for further evaluation in a second phase. In this phase, those who screened positive during the first phase were examined by a research physician specialized in neurological disorders. A structured clinical work-up, including the motor examination of the Unified Parkinson's Disease Rating Scale (UPDRS), a neurologic examination, and standardized history taking, was used to establish the diagnosis and classification of parkinsonism.

Possible prevalent cases were also identified during follow–up through continuous monitoring of participants medical files and pharmacy records. For all suspected cases of parkinsonism, the complete medical records (including letters from medical records of specialists and general practitioners) were studied and case reports were compiled to establish the subtype of parkinsonism and the degree of certainty in the diagnosis.

The final diagnoses were adjudicated in a consensus panel led by an experienced neurologist. PD was defined in a person with parkinsonism by exclusion of all other possible causes of parkinsonism. (24)
