# Supplementary material for: Sex-specific patterns and lifetime risk of multimorbidity in the general population: a 23-year prospective cohort study
Source: BMC Med. 2022 Sep 8;20:304. doi: 10.1186/s12916-022-02487-x (PMC9454172; doi:10.1186/s12916-022-02487-x)
Supplement: Supplementary file 2 — Additional file 2. Sensitivity analysis. Results of the five sensitivity analyses presented. [file 12916_2022_2487_MOESM2_ESM.docx]

**Additional file 2: Sensitivity analysis**

Additional file for article Velek, P, Luik AI, Brusselle GGO, *Sex-specific patterns and lifetime risk of multimorbidity in the general population: a 23-year prospective cohort study*

### **Sensitivity analyses**

#### **Sensitivity analysis 1**

Grouping dementia and depression together as mental health diseases did not change the difference between men and women: whereas men had 39.5% (95% CI: 36.6-42.5%) lifetime risk of multimorbidity with only physical diseases, the same risk among women was 25.1% (95% CI: 22.8-27.6%). Conversely, the lifetime risk of multimorbidity with mental health diseases was higher among women (40.0%, 95% CI: 37.3-42.7%) than in men (26.5%, 95% CI: 23.9-29.4%).

**
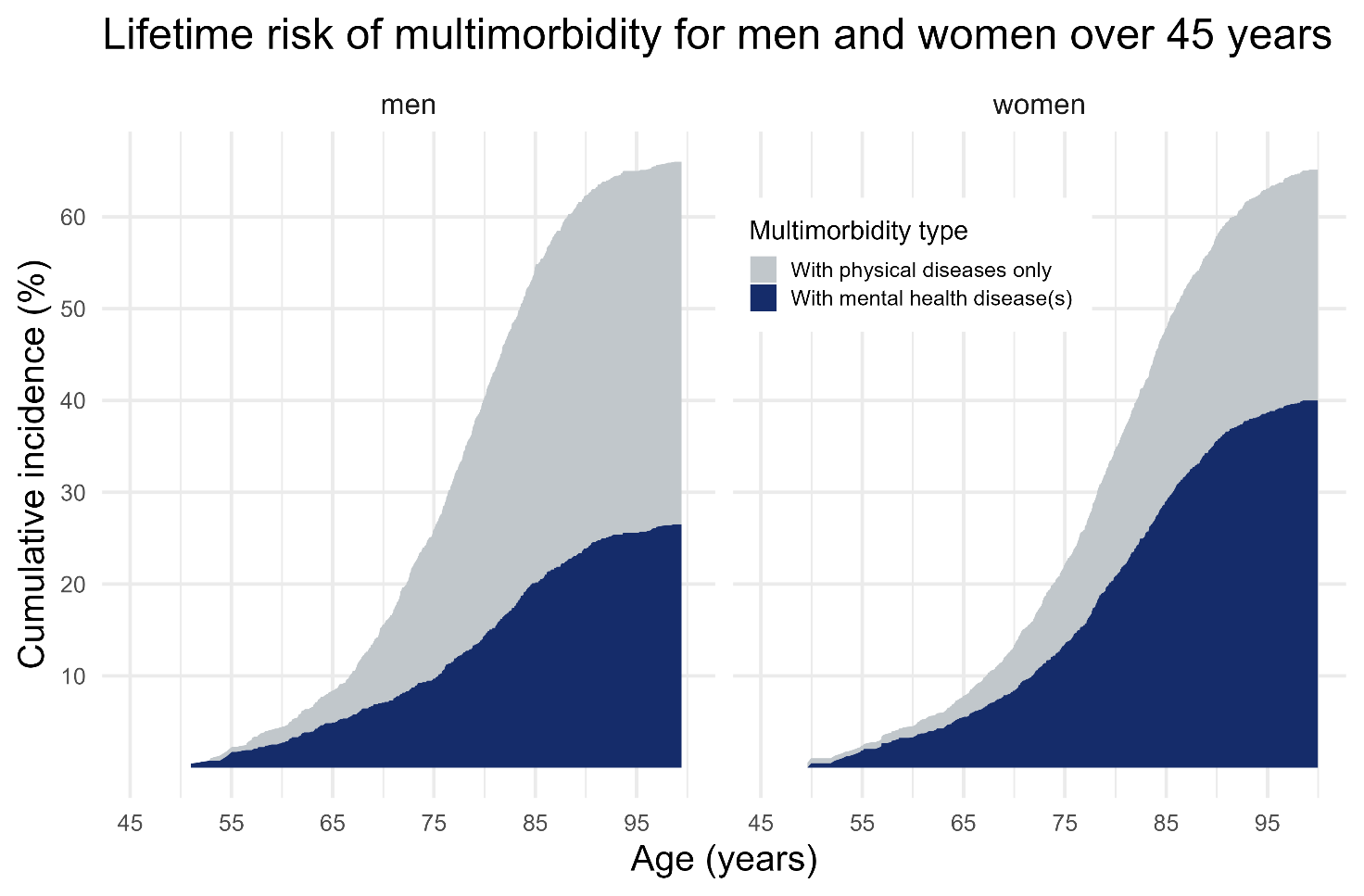
**

**Figure A1**: Lifetime risk of multimorbidity for men and women over 45 years with disease grouping according to ICD-10 classification. Any combination of diseases involving depression or dementia were classified as multimorbidity with mental health disease(s), any other combination were classified as multimorbidity with physical diseases.

#### **Sensitivity analysis 2**

Grouping stroke, parkinsonism and dementia as neurological diseases did not change the results substantially. The risk of somatic concordant multimorbidity increased to 6.3% (95% CI: 5.0-7.9%) for men and to 7.0 % (95% CI: 58-8.5%) for women. The risk of somatic discordant multimorbidity decreased to 42.2% (95% CI: 39.2-45.1%) for men and to 27.2% (95% CI: 24.8-29.7%) for women; the risk of somatic-psychosocial multimorbidity did not change.


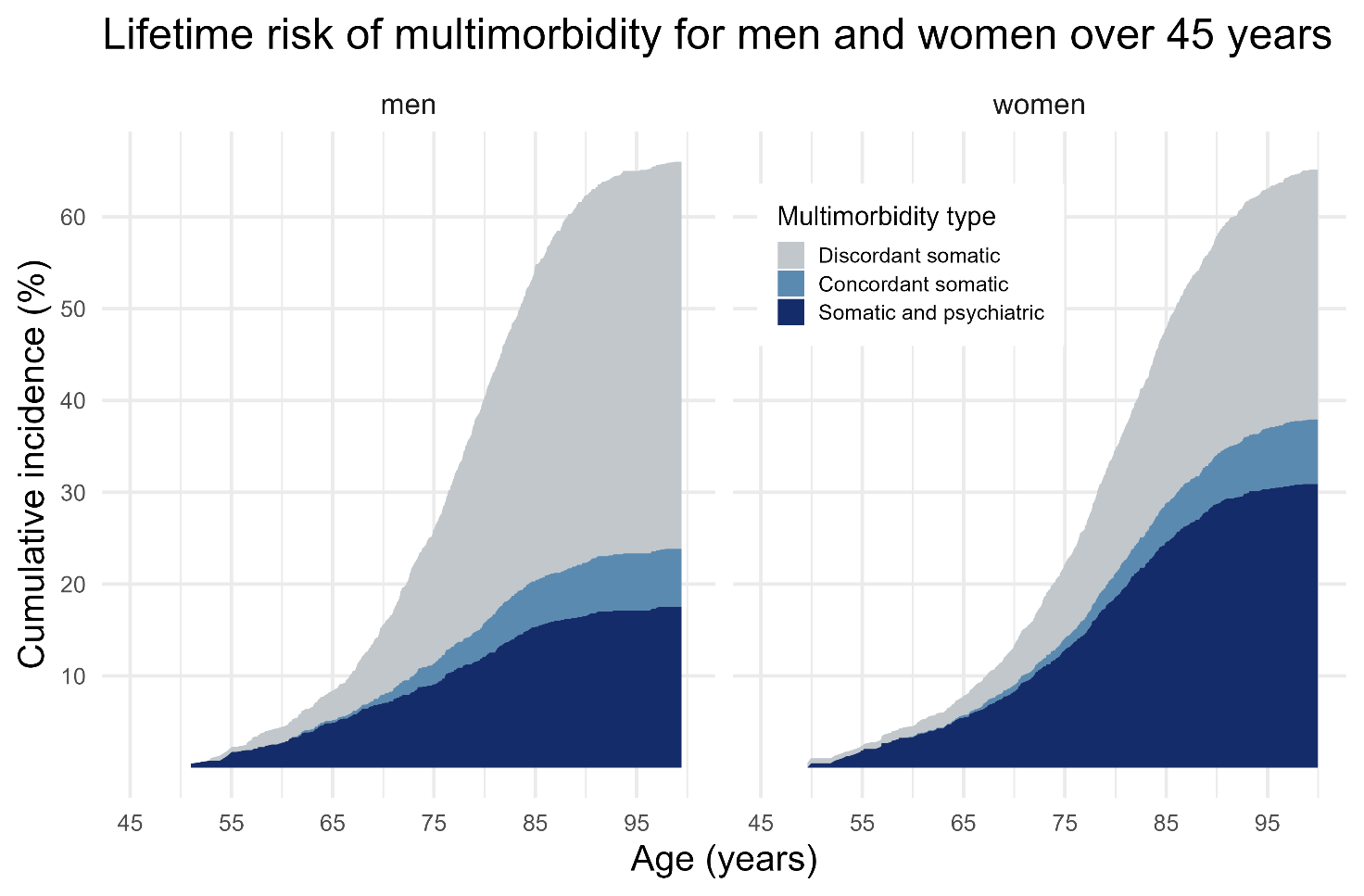


**Figure A2**: Lifetime risk of multimorbidity for men and women over 45 years with dementia, parkinsonism and stroke grouped together as neurological diseases. Any combination of dementia, parkinsonism and stroke were classified as concordant multimorbidity, any other combination were classified the same as in the main analysis.

#### **Sensitivity analysis 3**

When treating cases of depressive symptoms as depression free, the population size grew from 6,094 to 6,493 participants, as participants with prevalent cases of depressive symptoms were added in. The lifetime risk of somatic-psychiatric multimorbidity dropped when analyses were limited to major depressive cases with clinically relevant depressive symptoms excluded. Yet, the lifetime risk involving depression remained twice as high for women compared to men (12.0% (10.3-13.9) for women vs 6.4% (5.0 – 8.1) for men). The overall risk of multimorbidity dropped more substantially for women than for men (61.2% (95% CI: 58.3-63.4%) for men and 55.7% (95% CI: 52.5-58.1%) for women). The risk of somatic discordant multimorbidity increased for both men and women, but was substantially higher for men (49.6%, 95% CI: 46.1-52.1%) than for women (39.1%, 95% CI: 37.2-41.8%).

**
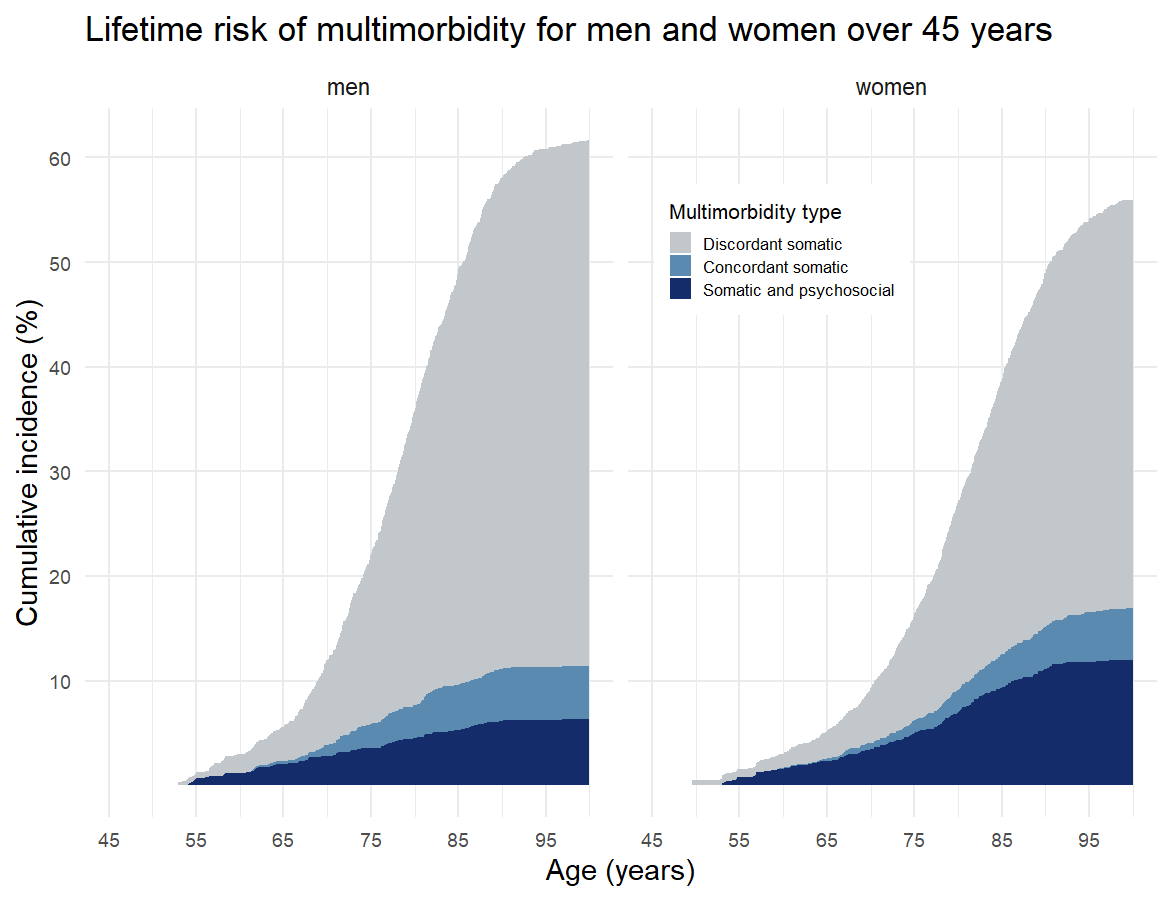
**

**Figure A3**: Lifetime risk of multimorbidity for men and women over 45 years. In this analysis, we considered only cases of depression that met the DSM-IV criteria.

#### **Sensitivity analysis 4**

Adding the third disease in chronological order changed the membership in multimorbidity class (from somatic-somatic to somatic-psychiatric multimorbidity) of 18 men (5.7% of those with somatic-somatic multimorbidity that had a third disease), and 23 women (10.3%).


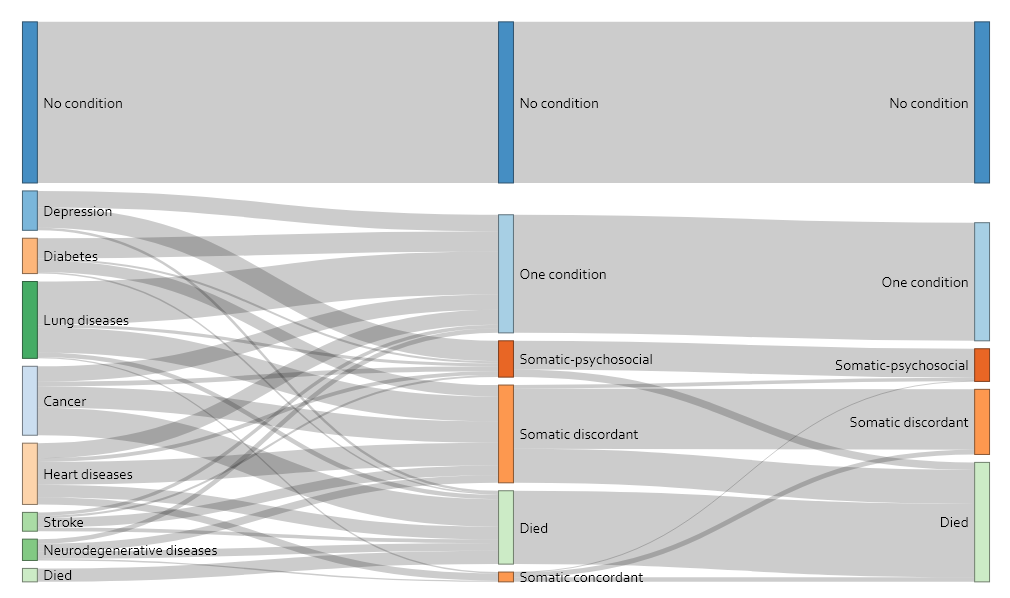
Men

Women


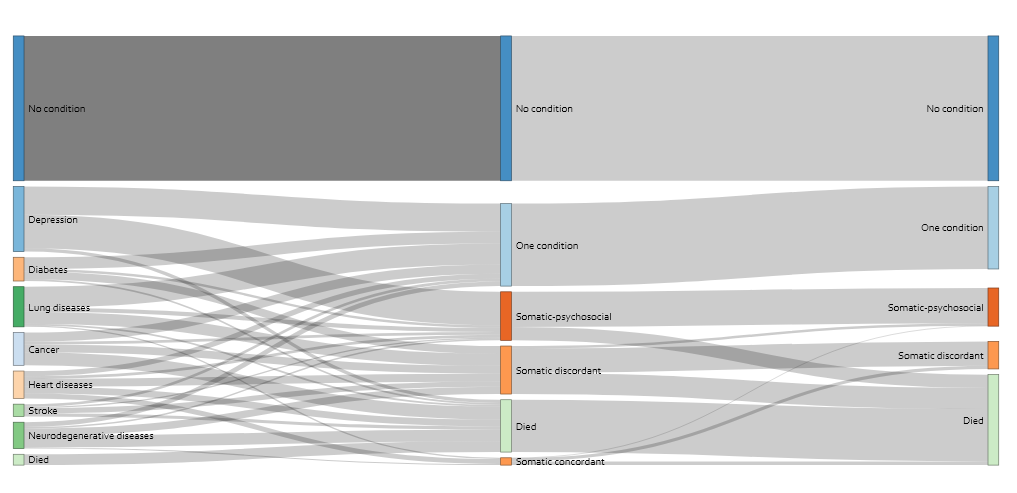
**Figure A4**: Disease trajectories from single disease to multimorbidity.
The columns represent the diagnosis of first three diseases in chronological order (from left to right). The height of the columns are the thickness of the stripes are proportional to the number of people with a particular disease. Neurodegenerative disease include Parkinsonism and Dementia, Heart disease include Coronary heart disease and Heart failure, Lung disease include COPD and Asthma. The diseases in the second and third diseases are grouped according to our definition on multimorbidity classes and show the change in multimorbidity membership classes when going from second to third disease in chronological order.

#### **Sensitivity analysis 5**

When included participants with the history of no more than one disease at baseline (total sample size n = 8,768), the overall risk of multimorbidity increased as expected. The overall lifetime risk of multimorbidity was 75.0 % (95 CI: 73.3-77.3%) for men and 73.0% (95% CI: 71.1 -74.9%) for woman (p-value for the sex difference 0.046). The relative sex difference in the estimated lifetime risk of multimorbidity involving psychiatric disease remained the same: 17.9% (95% CI: 16.4-19.7%) for men and 33.9% (95% CI: 32.1-35.9%) for women (p-value < 0.001). The lifetime risk of multimorbidity with two somatic discordant diseases was (51.7% (95% CI: 49.5-54.0%) for men vs. 35.5% (95% CI: 32.5-36.5%) for women (p-value < 0.001). The lifetime risk of somatic concordant multimorbidity remained relatively low (5.6% (95% CI: 4.6-6.7%) for men and 4.6% (95% CI: 3.8-5.5%) for women, p-value for sex difference 0.07).

**
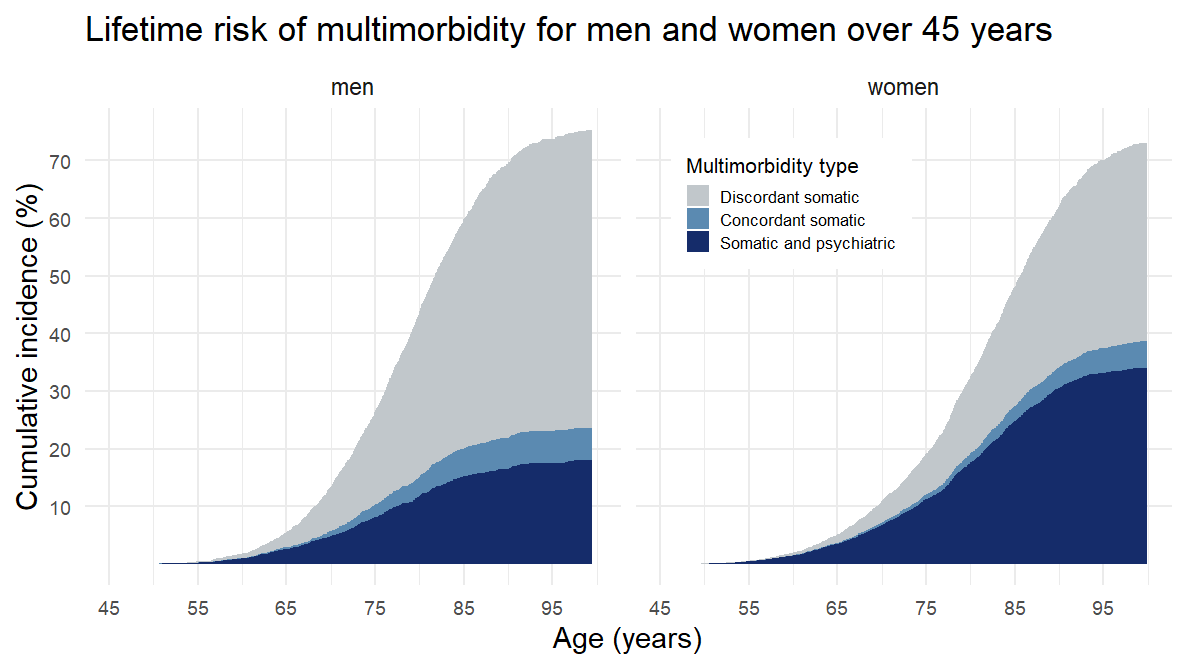
**

**Figure A5**: Lifetime risk of multimorbidity for men and women over 45 years. In this analysis, we included also participants with one prevalent disease at baseline (n = 8,836
