# Supplementary material for: Sex-specific patterns and lifetime risk of multimorbidity in the general population: a 23-year prospective cohort study
Source: BMC Med. 2022 Sep 8;20:304. doi: 10.1186/s12916-022-02487-x (PMC9454172; doi:10.1186/s12916-022-02487-x)
Supplement: Supplementary file 3 — Additional file 3. Population characteristics of excluded participants. [file 12916_2022_2487_MOESM3_ESM.docx]

**Additional file 3: Population characteristics of excluded participants**

Additional file for article Velek, P, Luik AI, Brusselle GGO, *Sex-specific patterns and lifetime risk of multimorbidity in the general population: a 23-year prospective cohort study*

### **Characteristics of excluded participants**

Table A1: Characteristics of excluded participants (all excluded participants)

| Characteristics | **All participants**  **(**n = 8,832**)** | **Men**  (n = 3,518, 39.8%**)** | **Women**  **(**n = 5,314, 60.2%**)** | **p-value for sex differences**^1^ |
| --- | --- | --- | --- | --- |
| **Age (years), median (IQR)** | 69.9 (61.3-79.2) | 68.4 (61.0-76.3) | 71.5 (61.4-81.2) | < 0.001 |
| **Marital status** | | | | < 0.001 |
| Living with partner | 4921 (62.3%) | 2613 (80.6%) | 2308 (49.6%) |  |
| Living without partner | 2976 (37.6%) | 627 (19.4%) | 2349 (50.4%) |  |
| **Education** | | | | < 0.001 |
| Primary | 2081 (24.7%) | 567 (16.5%) | 1514 (30.3%) |  |
| Lower secondary | 3308 (39.2%) | 1061 (30.8%) | 2247 (45.0%) |  |
| Further secondary | 2096 (24.8.5%) | 1201 (34.9%) | 895 (17.9%) |  |
| Higher | 950 (11.3%) | 612 (17.8%) | 338 (6.8%) |  |
| **Smoking status** | | | | < 0.001 |
| Current | 2056 (24.3%) | 1055 (30.7%) | 1001 (20.0%) |  |
| Former | 3626 (43.0%) | 2034 (59.2%) | 1592 (31.8%) |  |
| Never | 2759 (32.7%) | 349 (10.2%) | 2410 (48.2%) |  |
| **Blood pressure** (mm Hg), median (IQR) | | | |  |
| Systolic | 141 (126-157) | 141 (126-156) | 142 (126-159) | 0.09 |
| Diastolic | 77 (69-85) | 78 (69-86) | 76 (68-85) | 0.001 |
| **Ancestry** | | | | 0.66 |
| European | 6351 (98.1%) | 2655 (98.2%) | 3696 (97.9%) |  |
| East-Asian | 84 (1.3%) | 31 (1.1%) | 53 (1.4%) |  |
| African | 32 (0.5%) | 13 (0.5%) | 19 (0.5%) |  |
| Admixed | 10 (0.2%) | 4 (0.1%) | 6 (0.2%) |  |

IQR: Interquartile range. There were 10.5% of missing data for marital status, 9.5% of missing data on education, 4.4% of missing data on smoking status, 0.9% missing data on blood pressure, and 26% of missing data on ancestry. Missing data were excluded when calculating the summary statistics.

1. p-values were calculated using Wilcoxon-Mann-Whitney rank sum for continuous variables (with the null hypothesis of equal medians); and Chi-squared test for categorical variables.

Table A2: Characteristics of excluded participants due to incomplete follow-up data

| Characteristics | **All participants**  **(**n = 5,019**)** | **Men**  (n = 1,854, 36.9%**)** | **Women**  **(**n = 3,165, 63.1%**)** | **p-value for sex differences**^1^ |
| --- | --- | --- | --- | --- |
| **Age (years), median (IQR)** | 74.3 (65.7-82.1.2) | 72.3 (65.1-79.4) | 75.7 (66.2-83.6) | < 0.001 |
| **Marital status** | | | | < 0.001 |
| Living with partner | 2,456 (54.5%) | 1,296 (76.6%) | 1,160 (41.1%) |  |
| Living without partner | 2,051 (45.5%) | 397 (23.4%) | 1,654 (58.7%) |  |
| **Education** | | | | < 0.001 |
| Primary | 1,502 (31.5%) | 395 (21.9%) | 1,107 (37.4%) |  |
| Lower secondary | 1,821 (38.2%) | 568 (31.5%) | 1,253 (42.3%) |  |
| Further secondary | 1,046 (22.0%) | 581 (32.2%) | 465 (15.7%) |  |
| Higher | 395 (8.29%) | 259 (14.4%) | 136 (4.6%) |  |
| **Smoking status** | | | | < 0.001 |
| Current | 1,209 (25.2%) | 619 (34.3%) | 590 (19.8%) |  |
| Former | 1,836 (38.3%) | 1,037 (57.5%) | 799 (26.8%) |  |
| Never | 1,744 (36.4%) | 148 (8.2%) | 1,596 (53.5%) |  |
| **Blood pressure** (mm Hg), median (IQR) | | | |  |
| Systolic | 144 (128-160) | 142 (126-158) | 145 (129-161) | < 0.001 |
| Diastolic | 76 (68-85) | 76 (67-85) | 75 (68-84) | 0.012 |
| **Ancestry** | | | | 0.54 |
| European | 3,198 (98.6%) | 1,237 (98.6%) | 1,961 (98.7%) |  |
| East-Asian | 34 (1.0%) | 12 (1.0%) | 22 (1.1%) |  |
| African | 9 (0.3%) | 5 (0.4%) | 4 (0.2%) |  |
| Admixed | 1 (0.03%) | 1 (0.1%) | 0 (0.0%) |  |

IQR: Interquartile range. There were 10.2% of missing data for marital status, 7.2% of missing data on education, 6.6% of missing data on smoking status, 24.1% missing data on blood pressure, and 54.8% of missing data on ancestry. Missing data were excluded when calculating the summary statistics.

1. p-values were calculated using Wilcoxon-Mann-Whitney rank sum for continuous variables (with the null hypothesis of equal medians); and Chi-squared test for categorical variables.

Table A3: Characteristics of excluded participants due to prevalent cases

| Characteristics | **All participants**  **(**n = 3,500**)** | **Men**  (n = 1,585, 45.2%**)** | **Women**  **(**n = 1,915, 54.7%**)** | **p-value for sex differences**^1^ |
| --- | --- | --- | --- | --- |
| **Age (years), median (IQR)** | 62.9 (58.4-71.7) | 59.0 (63.5-70.8) | 58.0 (62.5-72.4) | 0.13 |
| **Marital status** | | | | < 0.001 |
| Living with partner | 2,371 (74.0%) | 1,275 (85.4%) | 1,096 (64.1%) |  |
| Living without partner | 833 (26.0%) | 218 (14.6%) | 615 (35.9%) |  |
| **Education** | | | | < 0.001 |
| Primary | 523 (15.0%) | 159 (10.1%) | 364 (19.1%) |  |
| Lower secondary | 1,424 (40.9%) | 475 (30.1%) | 949 (49.8%) |  |
| Further secondary | 1,010 (29.0%) | 606 (38.4%) | 404 (21.2%) |  |
| Higher | 528 (15.2%) | 339 (21.5%) | 189 (9.9%) |  |
| **Smoking status** | | | | < 0.001 |
| Current | 804 (23.2%) | 416 (26.4%) | 388 (20.5%) |  |
| Former | 1,721 (49.7%) | 968 (61.5%) | 753 (39.9%) |  |
| Never | 939 (27.1%) | 191 (12.1%) | 748 (39.6%) |  |
| **Blood pressure** (mm Hg), median (IQR) | | | |  |
| Systolic | 139 (125-154) | 140 (127-155) | 138 (123-154) | 0.006 |
| Diastolic | 78 (70-87) | 79 (71-88) | 78 (70-86) | < 0.0001 |
| **Ancestry** | | | | 0.30 |
| European | 3,036 (97.5%) | 1,389 (98.0%) | 1,647 (97.1%) |  |
| East-Asian | 48 (1.5%) | 18 (1.3%) | 30 (1.8%) |  |
| African | 23 (0.7%) | 8 (0.6%) | 15 (0.9%) |  |
| Admixed | 7 (0.2%) | 2 (0.1%) | 5 (0.3%) |  |

IQR: Interquartile range. There were 8.5% of missing data for marital status, 0.4% of missing data on education, 1.0 % of missing data on smoking status, 0.2% missing data on blood pressure, and 12.4% of missing data on ancestry. Missing data were excluded when calculating the summary statistics.

1. p-values were calculated using Wilcoxon-Mann-Whitney rank sum for continuous variables (with the null hypothesis of equal medians); and Chi-squared test for categorical variables.
